# Case report: Case series of urinary retention in young adults with severe autism hospitalized for behavioral crisis

**DOI:** 10.3389/fpsyt.2025.1570436

**Published:** 2025-05-13

**Authors:** Leslie Nollace, Drolapas Panagiotis, Josephine Convertini, Nuno Grilo, Nicolas Ansermot, Vincent Guinchat

**Affiliations:** ^1^ Psychiatric Section of Mental Development, Department of Psychiatry, Lausanne University Hospital and University of Lausanne, Lausanne, Switzerland; ^2^ Department of Psychiatry, Lausanne University Hospital, Lausanne, Switzerland; ^3^ Department of Urology, Lausanne University Hospital, Lausanne, Switzerland; ^4^ Unit of Pharmacogenetics and Clinical Psychopharmacology, Center for Psychiatric Neuroscience, Department of Psychiatry, Lausanne University Hospital, University of Lausanne, Prilly, Switzerland

**Keywords:** autism, urinary retention, behavioral crisis, challenging behaviors, physical comorbidity

## Abstract

**Background:**

Behavioral regressions in low verbal patients with severe autism constitute a dramatic challenge for clinicians. A physical comorbidity burden is often involved but difficult to evidence.

**Aim:**

We present five cases of patients under 30 years old (three men and two women), recently hospitalized in a specialized multidisciplinary inpatient unit, settled in Lausanne University Hospital, and for which at some point, a urinary retention contributed to the constitution of a complex behavioral picture.

**Methods:**

For each patient, we report the individual risk factors, clinical presentation and the conditions for making the diagnosis.

**Results:**

As the usual guidelines for screening, management, and follow-up of urinary retentions are irrelevant in this population, we provide and discuss some recommendations: limitation of anticholinergic burden, strict application of the protocol for going to the toilet with training protocol, regularization of intestinal transit, daily bladder-scan control, and eventually use of Tamsulosin hydrochloride. These recommendations significantly improved the urinary status of our patients.

**Conclusion:**

We conclude that chronic urinary retention is probably a recurrent and unrecognized feature in many young adults with autism and challenging behaviors, reflecting the long-term impact of iatrogenic medication and requiring a specific attention.

## 1 Introduction

Autism spectrum disorder (ASD) is a neurodevelopmental disorder, defined by persistent deficits in social communication and interaction and restricted, repetitive behaviors, interests, or activities (1). Even if they are not constitutive of its definition, challenging behaviors are often observed in this population: self-injurious behaviors, hetero-aggressivity, major disruptive behaviors, or catatonia can sometimes lead to severe behavioral regression, especially during adolescence. Their treatment is a complex endeavor, especially in severe ASD and/or intellectual disability (ID) (2, 3). Due to their severity (intensity, frequency, duration, and/or localization), these behaviors often result in dramatic and sometimes even life-threatening conditions. Causes are extremely diverse but include comorbid medical conditions, either organic or psychiatric (2, 4). Such conditions are often unrecognized because patients cannot communicate or locate their discomfort. It often leads to long-term misuse of psychotropic drugs with iatrogenic consequences. A comprehensive approach is necessary not only to alleviate the impact of challenging behaviors on patients and their caregivers but also to orientate towards an underlying physical condition that needs to be treated. The most complex clinical picture may benefit from hospitalizations in multidisciplinary units dedicated to challenging behaviors. In the present study, we present the unexpected discovery of urinary retention (UR) in hospitalized patients not otherwise known for urinary disorders. Urinary retention (UR) is common in older male subjects, particularly with prostatic obstruction, but is rarely reported in young subjects. UR is defined by the inability to urinate, despite bladder repletion. It is generally classified into “acute” or “chronic” urinary retention. Acute urinary retention (AUR) is the sudden inability to void, usually associated with bloating, severe urgency, general distress, lower abdominal distension, and suprapubic pain (5). The American Urological Association (AUA) defines chronic urinary retention (CUR) by a post-mictional residue >300 ml documented by two paraclinical examinations 6 months apart. Due to the retention, patients may feel incomplete emptying, overflow incontinence, and symptoms of weak or intermittent stream. The symptoms associated with these urinary voiding disorders present inter-individual variability and encompass a spectrum including dysuria, pollakiuria, urinary urgency, with or without incontinence, and recurrent urinary tract infections. Prolonged persistence of these conditions can potentially give rise to complications, notably dysfunction of the upper urinary tract with irreversible sequelae. These voiding disorders may emanate from infravesical obstruction (mainly benign prostatic hyperplasia, and acute prostatitis but also prostate cancer and fecaloma) or from alteration of the bladder contraction (neurogenic dysfunction, peripheral or central, or iatrogenic). Normal functional bladder capacity in adults rates from approximately 300 ml to 400 ml. Normal micturition occurs when bladder contraction is coordinated with relaxation of the urethral sphincter. The central nervous system inhibits urination until the appropriate moment and coordinates the onset and termination of urination. Because of the complex mechanism of micturition, many drugs can interact with the micturition pathway via different modes of action (3, 6–9). AUR has been associated especially with the use of drugs that possess anticholinergic effects (quetiapine, promazine, and clozapine), different degrees of anticholinergic burden being described (10, 11). This anticholinergic effect is caused by a blockade of the parasympathetic pathway, involved in contraction of the detrusor muscle of the bladder. Some prescriptions such as risperidone involve, in particular, the central dopamino-serotoninergic micturition control (3, 12, 13). Co-prescription with antidepressants such as fluoxetine can also increase the risk for UR. The literature linking urinary retention, ASD, and challenging behaviors is scarce. Most studies focused on the frequency of enuretic symptoms (14, 15) and the concomitant bowel dysfunction, and one study reported an association with mood and anxiety disorders (16). Some authors suggested guidelines for the management and recognition of bladder bowel dysfunction (17, 18). Recently, in a national survey including 502 ASD children, low urinary tract symptoms were reported by parents in 77.1%, storage symptoms in 51.4%, and voiding symptoms in 60.6% (19). If low urinary tract symptoms seem to affect the vast majority of young ASD subjects, methodological concerns make it challenging to uncover the true extent of the problem. We assume that UR may be a recurrent and unrecognized feature in many young adults with autism and challenging behavior, which often reflects long-term iatrogenic effects and requires considerable attention.

In this report, we present five young adults with autism and severe intellectual disability, hospitalized for acute behavioral regression including severe aggressivity, who developed at least one episode of UR during their inpatient stay, as evidenced through a systematic screening procedure. Previous ASD diagnosis was supported for all five patients during childhood by a clinician with many years of experience in working with autistic patients and confirmed during the inpatient stay in our specialized multidisciplinary unit.

## 2 Case series

Our descriptions focus on the circumstances of the detection of urinary retention in five complex cases. For the sake of brevity, clinical characteristics are presented in Table 1 (see **Supplementary Table S1**) including the retained diagnosis underlying the behavioral regression. Specific information regarding their urinary conditions is summarized in Table 2 (see **Supplementary Table S2**).

The first case is a 20-year-old man, known for autism, moderate intellectual disability, and behavioral disorders (severe self-injurious behaviors and severe hetero-aggressivity). After occasional behavioral problems as a child, a change in his behavior appeared during adolescence with transition to severe aggressiveness towards himself and caregivers. He received treatment based on antipsychotics since childhood (first treatment at the age of 9 with risperidone). The patient’s mother described several episodes of pollakiuria at the age of 13–14 without any further urinary examinations. During hospitalization, we observed, several times a week, episodes during which the patient had a sad face or was crying, with a flexed trunk position consistent with pain in the region of the abdomen. Auto-aggressive challenging behaviors (punches to his abdomen, his thighs, and his head) and severe hetero-aggressive challenging behaviors were often associated with these episodes. Due to the patient’s irregular intestinal transit, we first considered a digestive hypothesis and set up a protocol of laxatives against constipation. We had to introduce clozapine for very severe resistant challenging behaviors. During a tantrum, we performed a bladder scan that showed a volume of urine of 500 ml. We also noted that the volume urinated by the patient during his micturition seemed very high (volume not quantified by our team initially, due to the patient’s lack of compliance). The patient underwent a consultation in the Urology Department. An ultrasound revealed bilateral pyelocalic ectasia and a bladder that appeared to show signs of hypertonic bladder, a bladder capacity of 1,300 ml. After voiding, the patient exhibited a post-void residual of 500 ml assessed via urinary catheterization. Urologists recommended an indwelling catheter for the long term. However, due to patient’s risk of severe agitation, the indwelling urethral catheter had to be removed after 1 h. Instead, timed voiding was implemented every 3 h during the day (caregivers and educators implemented a strict protocol to encourage the patient to urinate in the toilet regularly), and patient was prescribed an alpha blocker (Tamsulosin hydrochloride 0.4 mg daily). A bladder scan was performed before and after urination, initially daily, then weekly, and in case of agitation. In the event of significant post-void residual, intermittent catheterization was performed. Throughout the rest of the hospitalization, we focused our efforts on reducing the drugs, which potentially increase the risk of UR. We maintained clozapine (350 mg daily) for the resistant comorbid psychotic disorders, looking for the minimum effective dose and reducing other drugs with anticholinergic effects. We also improved transit with a stricter laxative protocol. Gradually, the episodes of UR have completely disappeared: after a few weeks, intermittent catheterization was no longer needed due to the absence of significant post-void residual. The patient is followed by the Urology Department after discharge.

The second case is a 27-year-old woman at admission, known for autism, severe intellectual disability, and behavioral disorders (severe self-injurious behaviors, severe hetero-aggressivity, and tantrums) since childhood, these episodes being even more present during the last year. She was transferred from another general psychiatric unit where she was isolated in a secured room. She had been taking antipsychotic medication since childhood (first treatment at the age of 6 with risperidone). The parents described recurrent urinary tract infections during the last years, treated by different antibiotics but without additional examinations. During the first 6 weeks of hospitalization, the patient showed a transient improvement with her challenging behaviors until suddenly extremely severe aggressive behaviors appeared towards her and others. She screamed, banged her head severely against the walls, bit her arms, threw objects violently on the floor, and bit and severely hit caregivers. She also characteristically threw water on her abdomen and her pubic area. At this moment, a CT scan was performed, which showed a UR (900 ml) with a thin-walled bladder and constipation. She finally managed to void her bladder spontaneously. In the following weeks, polypharmacy was reduced, and the risk–benefit assessment of every new treatment was weighted with regard to UR risk. We finally had to introduce clozapine (300 mg daily) for treating the resistant comorbid psychotic disorders, reducing other drugs with anticholinergic effect. The medical team and caregivers also followed the same procedure as for patient 1, that is, accompanying the patient to the toilet every 3 h to prevent recurrence of UR and performing a bladder scan before and after urination, several times a week, and in case of agitation. We have regularized bowel movements thanks to a stricter laxative protocol. The patient did not present recurrences of UR or urinary infections during hospitalization. The patient is now followed in an outpatient setting by the Urology Department.

The next case report is a 25-year-old man with Down syndrome, severe intellectual disability, and autism, known for extremely severe self-injurious behaviors since his early childhood. He suffered from total loss of vision in one eye and significant loss of vision of the other eye, following self-inflicted trauma. He required mechanical restraints in his institution for preserving its visual outcome. He had been receiving antipsychotic treatment since childhood (first treatment at the age of nine with risperidone). The parents mentioned a diurnal urinary incontinence and nocturnal enuresis for several years. During his hospitalization, he developed increased aggressivity towards himself and others (blows with his hands to his face, in particular his eyes, and bites on caregivers) and increase of screams and tears. An absence of urine for more than 6 h has been the warning sign: the bladder scan confirmed the AUR (volume of 650 ml), resulting in drainage in the emergency department of 1,000 ml of urine by catheterization. A context of increased olanzapine dose and introduction of clobazam was present. The indwelling urethral catheter was recommended for 1 week but has been removed after 48 h due to patient discomfort with agitation. We have implemented the accompanying procedure, with particular attention to the fact that the patient urinates sufficiently often, every 3–4 h during the day at least. We also checked, daily then several times a week, the bladder scan. We stopped clobazam. Olanzapine could not be switched, but dosage was reduced. We introduced transiently an alpha blocker (Tamsulosin hydrochloride, 0.4 mg per day). The micturition problems have thus been solved. The patient was assessed by the urology team. Ultrasound revealed a complete emptying of the bladder, without residue after micturition. Tamsulosin hydrochloride was stopped without recurrence UR. We have highlighted, thanks to characterization of the cytochrome phenotype, that the patient was a fast metabolizer for clobazam (CYP3A activity accelerated), resulting in accumulation of the active metabolite of clobazam (norclobazam). Co-prescription of esomeprazole, which is a CYP2C19 inhibitor, also slowed the elimination of this active metabolite and increased the risk of its accumulation.

Case 4 is a 25-year-old man, known for autism, severe intellectual disability, and abnormal movements finally assigned to tardive dyskinesia. During the last years, he had developed multiple episodes of UR, which led him to the Emergency Department for catheterization, with major difficulties in managing these consultations for himself, for the emergency teams, and for his educators because of his lack of compliance with severe motor agitation. He had antipsychotic treatment since childhood (first treatment at the age of 9 with risperidone). We decided to introduce an alpha blocker (Tamsulosin hydrochloride 0.4 mg daily). Despite this medication, the patient presented multiple episodes of UR. During these episodes, we noticed that he was trying to undress himself, lowered his pants, and showed and touched his penis while trying to urinate. He presented a discomfort with a tense face, auto-aggressivity towards his abdomen or his head (abdomen pinches and punches to his head), and was unable to urinate for more than 6 h; sometimes, he also only urinated very small volumes of urine on the floor, most likely from overflow, considering that the patient previously urinated in the toilet. With the aid of the bladder scan, the crew of the department noticed a bladder volume approximately 700 ml during these episodes. These UR episodes required intermittent catheterization because the patient was unable to empty his bladder despite our encouragement. After catheterization, we rapidly noticed a relief and a better behavior of the patient. We observed that UR was systematically linked to episodes of constipation. We also suspected, in view of these episodes of repeated UR, the development of a neurogenic bladder, based on the context of neurological disorder with abnormal movements and that the patient was medicated by psychotropic drugs, in particular long-term use of antipsychotics. We increased our vigilance regarding bowel movements by implementing a very strict laxative protocol with several daily treatments and laxatives added if needed. We reduced most of the drugs potentially increasing the risk of UR, but we also had to introduce clozapine (100 mg daily) to alleviate tardive dyskinesia that was too impairing. We encouraged the patient to urinate more frequently. It was a complex task due to the severe intellectual deficit of this non-verbal patient, but the team worked to promote moments of relaxation during which we noticed that he could urinate. We also monitored rigorously the frequency of urination and performed a bladder-scan daily (before and after urination) or in case of agitation. After urology evaluation, to rule out a neurogenic dysfunction of the lower urinary tract, urodynamic assessment and cystoscopy were suggested. However, the realization of these exams currently seems difficult due to the behavioral particularities and patient’s intellectual deficit. An ultrasound showed a bladder with no post-micturition residue after uroflowmetry and without pyelocalic dilatation. The voiding symptoms have improved significantly. It was no longer necessary to perform intermittent catheterization. Rare episodes of UR reappeared punctually in case of recurrence of constipation but with spontaneous evacuation of urine and no significant post-void residual.

Last but not the least is a case of an 18-year-old man known for autism and severe intellectual disability, presenting extremely severe self-injurious behaviors and hetero-aggressivity (banging his head against the walls, bites of his arms, blows with hands and legs, and bites to caregivers). He was transferred to our unit after 6 weeks of hospitalization in general psychiatry enduring isolation in a secured room. His challenging behaviors were known from childhood but had severely increased in the last 6 months. He also had antipsychotic treatment since childhood (first treatment at the age of nine with risperidone). There is no medical history of urinary disorders according to the medical staff of the institution and his parents before the hospitalization in our unit. Since the first days of hospitalization, we discovered a fracture of his right clavicle, which required surgery. Tramadol analgesic treatment was introduced before the surgery (200–300 mg daily). This young patient developed an episode of UR of a volume of 850 ml. This episode was detected by a bladder scan carried out due to our experience with the previous cases described in contexts of agitation with no obvious environmental triggering factor, especially for this patient with polymedication potentially at risk for UR and with severe persistent aggressivity, which may be indicative of pain. At the time of the bladder scan, the patient had not urinated for at least half a day. The total emission of urine was spontaneous after request from caregivers to urinate. UR was no longer identified with the help of the unit’s caregivers, which repeatedly stimulated the patient to urinate, performing bladder scan several times a week (before and after urination) and ensuring a strict laxative protocol to prevent constipation.

In these five patients, renal function was preserved on biological blood tests.

All but one patient evolved very favorably from a behavioral point of view with a reduction in self- and hetero-aggressive challenging behaviors. The favorable evolution in terms of UR certainly contributed to this evolution.

## 3 Discussion

We present our experience in our new adult inpatient unit dedicated to severe autistic subjects with behavioral regressions and located in Lausanne. Between January 2022 and January 2023, when focusing on our youngest patients (between 18 and 27 years old), we report that 5 out of 11 (45%) had at least one episode of UR during their inpatient stay. As such manifestations are well established in elderly people or older patients with a long period of psychiatric follow-up (3, 20, 21), we were very surprised to detect them so frequently and with such high volumes of urine retention (700–1,300 ml) among this young age group, with individuals not otherwise known for congenital or chronic urinary pathologies before admission.

As a comparison, De Waal et al. (22) used a portable bladder scan to screen unnoticed post-void residual urine volume (>150ml) in 346 people with moderate to severe intellectual disabilities and identified UR in 8.7% subject. The overrepresentation of UR diagnoses in a context of behavioral regression for ASD patients deserves to be studied in more depth and treated in conjunction with psychiatric care.

The long-term management and evolution of such urinary symptoms in the population with intellectual disability and autism is uncertain and worrying. UR is associated to gradual urine accumulation, bladder distension, and increase in the intravesical pressure. Episodes of UR, or incontinence by overflow, can lead to chronic UR with possible urinary tract infections, cystitis, or pyelonephritis, due to reflux of urine towards the kidneys. Complicated urinary tract infections may cause loss of kidney parenchyma, impairing renal function and increasing the likelihood of chronic renal failure. Due to the lack of compliance of our patients, any indication of an indwelling catheter will be inapplicable. The evolution of the patients in this series, on the urinary and behavioral level, and consequently the quality of life has been favorable by implementing alternative measures. The advantage of these case reports is the ability to observe and provide solutions for urological pathologies, which act jointly on the risk of urinary complications and the challenging behaviors.

The lack of communication among our group of patients, autistic and non-verbal, poses difficulties for the health professionals to recognize or understand behaviors underlined by UR. Long-term stay in the specialized unit characteristics offers an access of evaluation and observation of the patient’s appearance, activity level, interactions with caregivers, and the daily and nocturnal behavior. Diagnosis was allowed by a systematic multidisciplinary clinical assessment. Retrospectively, this screening was not orientated by a recurrent specific behavioral correlate. However, we have identified behavioral patterns suggestive of UR (see **Supplementary Table S3**). The delay between two micturitions may have been the only consistent clinical cue. A prolonged delay between two micturitions (more than 6 h between two micturitions during the day) is easy for educators and caregivers to spot and with a good level of specificity. The fact that the urinary evacuation is realized by urinary incontinence (by overflow) may be difficult to evidence in case of primary enuresis.

In the present study, each clinical situation was unique in its complexity, but evidence of a urinary condition was concomitant, among the patients in this series, with the prescription of an antipsychotic drug that possesses anticholinergic effects (not necessarily a new introduction). A review from (13) identifies the relationship with the effects of behavioral pharmacotherapy on bladder function and especially in adults with behavioral disorders. Antipsychotics are currently used to treat ASD-associated comorbidities, such as schizophrenia spectrum disorders and behavior disorders. A systemic review by Pillay et al. suggested that they may also be useful in improving some core symptoms, particularly stereotyped behaviors, and narrow interests (23, 24). Currently, there is no evidence that any antipsychotic is more effective than others. Anyway, only risperidone in Switzerland, and risperidone and olanzapine in the USA, are allowed for treating irritability in ASD. In individual situations, an off-label prescription of other antipsychotics is justified when targeting a better effect profile. Clozapine is known for its better efficiency on resisting symptoms in schizophrenia. In our experience and in accordance with recent literature articles (25), it may also be true for resisting challenging behaviors in ASD. In four patients of our case series, it reduced the failure rates and improved very significantly the quality of life in reducing hyperactivity, irritability, and compulsions, and in increasing a better social communication and global functioning. Nevertheless, as for olanzapine, its action on the parasympathetic pathway via anticholinergic effects may impair the contraction of the detrusor muscle and therefore requires a cautious use. Our study suggests not to prohibit its prescription in patients with UR but, in case of use of this molecule, to maintain a high level of vigilance concerning the regularity of the patient’s urination (clinically and, if possible at least initially, with bladder scan) and to reduce as much as possible the other daily treatments with high anticholinergic burden (10, 11). In addition to the risk of UR under anticholinergic medication, a wide range of non-anticholinergic medications have effects on the bladder and urinary symptoms. In particular, in our series, prior long-term use of risperidone had probably interfered with central serotoninergic and dopaminergic mechanisms of control of the micturition reflex, resulting in an increased risk of UR (3, 13). In case of use of analgesic drugs, the treatment of pain with opiates or opioid analogues also decreases the sensation of bladder fullness by partially inhibiting the parasympathetic nerves that innervate the bladder and increase the tonus of the sphincter of the urinary bladder via sympathetic overstimulation, which leads to increased resistance in the outflow tract of the bladder (3).

Among the patients in our study, another parameter concomitant with UR was constipation. Dysfunctional voiding is one feature of bladder bowel dysfunction, which may lead to serious complications at older age (19). Its recognition and management through a multidimensional approach is crucial (18). Note that the association between UR and the dilation of the distal colon is bidirectional. The effects of a dilated rectum irritate the vesical trigone, enclose the posterior wall of the bladder, and obstruct the urethra. Rectal retention of feces could also lead to involuntary contraction of the pelvic floor muscles and the external anal sphincter, making bladder emptying difficult. Alternatively, a severe bladder dilatation can exert a mechanical compression on the intestinal loops, maintaining a vicious circle, leading to chronic impairment. Anticholinergic effect can also impact the UR and constipation simultaneously, and many risk factors for constipation had been documented in ASD (26–28).

A central dysfunction or an anatomical difference may also be evidenced in some forms of syndromic ASD such as Smith–Lemli–Opitz syndrome (29) or Cornelia de Lange syndrome (30). Nevertheless, for most ASD children, lower urinary tract and bowel dysfunctions may be due to many complex factors: maturational delay of bladder function, lack of recognition of or responding to a sensation, cognitive rigidities, and psychosocial stressors. Finally, it is likely that UR and constipation are linked via a common biological pathway through comorbid conditions such as dysautonomia (31, 32) and mood or anxiety disorders (16).

In our patient’s history, behavioral symptoms could vary in type and severity over time, with periods of worsening and remission. Therefore, we hypothesize that before admission to the hospital, some transient crisis period may have been caused or worsened by undetected AUR. Repeated episodes, with gradual urine accumulation and subsequent stasis, may have insidiously increased the vesical volume. We assume that UR was not an initial major risk factor for behavioral regression, but could be at least an insidious aggravating factor, which was more obviously symptomatic in the context of behavioral crises. We hypothesize that the development of recurrent AUR was facilitated by cumulative factors during development. The iatrogenic effect of the antipsychotic drugs may have acted as a precipitating factor of an underlying vulnerability. The voiding dysfunction may be initially mainly functional, through chronic stress or anxiety (33). Finally, we suggest that aspects such as poor attention to urination in context of behavioral disorders may be contributing to the bladder dysfunction. During episodes of severe behavioral deterioration with agitation and even aggressiveness, the patient and caregivers themselves are probably less attentive to the rhythm of urination and signs of the need to urinate.

The limitation of this case series is the lack of supplementary exams such as voiding cystourethrography that is necessary to detect cystoureteral reflux and evaluate the bladder neck and urethra for obstruction or other anatomic abnormalities. In addition, uro-flowmetry measures the volume of urine excreted in a single urination, and multichannel urodynamic test can evaluate bladder/urethral innervation and bladder voiding function during bladder filling and emptying. In addition, rectal ultrasound with measurement of the diameter and identification of colonic impaction would have been useful to evidence bowel bladder dysfunction. Anyway, such tests require catheterization of the bladder and rectum, so cooperation of the patient and caregiver is important.

## 4 Recommendation for management

We recommend a systematic screening of UR in case of behavioral crisis, even for young subjects. In the context of repeated or severe agitation, the use of the bladder scan should therefore be generalized to control urinary volume. The bladder scan is a useful instrument in our service and is recommended for all the departments that hospitalize patients with behavior disorders. It is a non-invasive portable device that provides an image and estimation of the volume of urine retained within the bladder: clinicians and nurses are able to evaluate the presence of UR correlated to a behavior disorder. A follow-up on the plan of the bladder and kidneys, at least biologically by blood tests (urea, creatinine, and clearance), and ideally by ultrasound, is warranted for patients with behavioral crisis, especially for patients with a history of UR and with persistence of iatrogenic risk factors. If UR is detected, either by chance or following an agitation, an overall assessment of the patient in the urology department is necessary for a specialized clinical evaluation (evaluation of the abdomen, back, genitalia, lower extremities, and neuromuscular function) (34), an ultrasound (kidney stones and hydronephrosis detection and comparison of pre- and post-voiding volume), and if possible a urodynamic assessment (detection of bladder hypo- or acontactility). Initial evaluation and clinical examination in the urology department will define follow-up on a 3-monthly, 6-monthly, or annual basis. The protocol for going to the toilet regularly is an effective practical measure to limit the risk of UR. Toilet training is a complex process that involves the cooperation of caretakers and educators. Various pictures and pictograms provide examples of training steps necessary for successful training. A reward may be offered for each successful step. A voiding diary may offer a urological evaluation. This diary provides information on the fluid consumption, timing and frequency of urination, incontinence episodes, discomfort, and behavior disorder. A regular voiding schedule (every 3–5 h) should be encouraged to improve bladder health and avoid urinary disorder. An empty bladder at night may be achieved with urination before bedtime. By this way, the caregivers can establish a micturition profile for each patient. Caregivers and educators should also pay particular attention to the frequency of urination of the patient in case of a behavioral crisis: they are probably less attentive to the rhythm of urination when dealing with a behavioral crisis, while this is particularly necessary in this context. Antipsychotics have negative effects on the bladder, although some are worse than others. These effects on bladder function can help clinicians to choose or to refrain from a group of medications, depending on the nature and severity of the underlying bladder dysfunction in a given patient with behavioral disorder. The choice of adjuvant medications (analgesics), long-term or even occasional treatment, should also take into account the risk benefit in urinary terms among this population. Keep in mind that transient prescriptions (benzodiazepines, antipsychotics, and even analgesics) can increase the risk of UR. Careful monitoring of urination allows introduction of clozapine, if not combined daily with another medication with high potency anticholinergic burden (10). A regular bowel function, thanks to the use of laxatives, is very important to succeed in controlling the risk of UR in our patients. We used different types of laxatives, often associated: osmotic agents, bulk-forming laxatives, stimulant laxatives, agonists of the serotonin 5-HT4 receptors, and agonists of the guanylate cyclase–C receptor. In case of increased constipation, we used rectal laxatives such as suppositories or enemas, stimulant laxatives, and increased doses of osmotic agents such as preparation used before digestive endoscopy. The application of Tamsulosin hydrochloride, an effective and well-tolerated treatment for patients with low urinary tract symptoms (LUTS), allows an empirical management, even in these cases of urinary disorders without obstructive symptoms due to prostatic hypertrophy (35).

## 5 Conclusion

Bladder dysfunction appears to be an underdiagnosed condition in ASD. Behavioral disorders in severe autistic people may mirror, at least for some of them, an early deleterious process of the urinary tract function. Our study highlights this phenomenon in young patients (under 30 years old). AUR is a painful event requiring immediate intervention, especially in this particular population characterized with autism and severe behavioral disorders. The association between the UR and the use of medication with anticholinergic burden is unequivocal; drug discontinuation or dose reduction should be considered. The strict application of the protocol for going to the toilet with training protocol, the limitation of anticholinergic burden (more generally of treatments increasing the risk of retention), the regularization of intestinal transit, and the use of Tamsulosin hydrochloride in men, associated with daily scanner bladder control initially, had brought some very encouraging results. The evolution was favorable among our patients studied, thanks to a caution management procedure, on the urinary and behavioral level. This small series of young adults with autism and severe intellectual disabilities should mention and always remind to the clinicians and psychiatrists the importance of solving a somatic problem.

## Data Availability

The original contributions presented in the study are included in the article/supplementary material. Further inquiries can be directed to the corresponding author.
