# Supplementary material for: Case report: Case series of urinary retention in young adults with severe autism hospitalized for behavioral crisis
Source: Front Psychiatry. 2025 May 13;16:1570436. doi: 10.3389/fpsyt.2025.1570436 (PMC12106420; doi:10.3389/fpsyt.2025.1570436)
Supplement: Supplementary file 1 [file Table1.docx]

2Supplementary Material

# Supplementary Figures and Tables

**Supplementary Table 1***.* Participant demographics, medications and clinical characteristics of behavioral crisis

| Sex, Age | Antipsychotic  drugs  chronic impregnation  (>5 years)  before hospitalization | Developmental  diagnosis and adaptive skills  Neurogenetic syndrome | Main symptoms  justifying  hospitalization | Medication –  hospital admission | Main risk factors for crisis determined at discharge | Medication –  hospital discharge |
| --- | --- | --- | --- | --- | --- | --- |
| Patient 1  Male  20 years old | Yes | -Autism  -Moderate intellectual disability  -Non-verbal  -KCNK9 + COL22A1  disorder (ongoing genetic research) | -Severe auto-aggressivity  (punches  to his head)  -Severe hetero-aggressivity  (grips, punches, headbutts) | -Guanfacine  -Lorazépam (AC score 1)  -Lévomépromazine (AC score 3)  -Diphenhydramine (AC score 3)  -Bipéridène (AC score 3)  *-Lévomépromazine (AC score 3)*  *-Doxylamine (AC score 3)* | -Adaptive environmental  -Psychiatric  (separation anxiety)  -Somatic (urinary retention, constipation) | -Clozapine (AC score 3)  -Mirtazapine (AC score 1)  -Diazepam (AC score 1)  -Tamsulosin  -Laxative (macrogol, prucalopride)  *-Diphenhydramine (AC score 3)*  *-Risperidone (AC score 1)*  *-Lorazepam (AC score 1)* |
| Patient 2  Female  27 years old | Yes | -Autism  -Severe intellectual disability  -Low verbal  - Ongoing genetic research | -Severe auto-aggressivity (headbutts  in the walls,  bites on her arms...)  -Severe hetero-aggressivity  (punches, headbutts) | -Aripiprazole (AC score 1)  -Clonazepam (AC score 1)  -Trazodone (AC score 1)  -Gabapentine  -Hydroxyzine (AC score 3)  *-Quetiapine (AC score 2)*  *-Clonazepam (AC score 1)* | -Adaptive environmental  -Psychiatric  (depression, psychosis)  - Somatic (gynecological issue, skin wounds, constipation) | -Clozapine (AC score 3)  -Fluoxetine (AC score 1)  - Laxative (macrogol , prucalopride)  *-Lorazépam (AC score 1)* |
| Patient 3  Male  25 years old | Yes | -Autism  -Severe intellectual disability  -Non-verbal  - Down syndrome | -Severe auto-aggressivity  (punches to his head mainly eyes)  -Severe hetero-aggressivity (punches, bites, headbutts) | -Olanzapine (AC score 2)  -Valproate de sodium (AC score 1)  *-Lorazepam (AC score 1)*  *-Clonidine*  *-Lévomépromazine (AC score 3)* | -Adaptive environmental  -Psychiatric  (bipolar disorder?)  -Somatic (gastritis, epilepsy, ophthalmological lesions, constipation, joint hypermobility) | -Olanzapine (AC score 2)  -Lamotrigine  -Lorazepam (AC score 1)  -Laxative (macrogol)  *-Lorazepam (AC score 1)*  *-Levomepromazine (AC score 3)* |
| Patient 4  Male  25 years old | Yes | -Autism  -Severe intellectual disability  -Non-verbal  - Variant of Unknown Significance (ongoing genetic research) | -Motor restlessness  -Insomnia | -Aripiprazole (AC score 1)  -Escitalopram (AC score 1)  -Valproate de sodium (AC score 1)  -Levomepromazine (AC score 3)  -Lorazepam AC score 1)  *-Lorazépam(AC score 1)* | -Adaptive environmental  -Iatrogenesis (tardive dyskinesia)  - Somatic (UR constipation,  otitis, joint hypermobility) | -Clozapine (AC score 3)  -Fluvoxamine (AC score 1)  -Tamsulosin  -Laxative (linaclotide, macrogol)  *-Lorazépam (AC score 1)* |
| Patient 5  Male  18 years old | Yes | -Autism  -Severe intellectual disability  -Low verbal  -CDK13 disorder (ongoing genetic research) | -Severe auto-aggressivity  (headbutts in the walls, bites)  -Severe hetero-aggressivity (headbutts, punches, bites) | -Pregabaline  -Sertraline (AC score 1)  -Diazepam (AC score 1)  *-Lévomépromazine (AC score 3)*  *-Diazepam (AC score 1)* | -Adaptive environmental  -Psychiatric  (depression, psychosis)  -Somatic (collarbone fracture, constipation, puberty, joint hypermobility) | - Clozapine (AC score 3)  - Naltrexone  - Fluoxetine (AC score 1)  - GnRH analogue  - Laxative (prucalopride)  *-Lorazepam (AC score 1)* |

*Reserves treatments are written in italics. Anticholinergic burden (CRIDECO Anticholinergic Load Scale, Ramos et al., 2022): low potency = AC score 1, medium potency = AC score 2, high potency = AC score 3.*

| Supplementary Table 2*.* Specific information regarding urinary condition for each participant |
| --- |

| Sex, Age, Weight | Clinical manifestations observed during UR | Recent changes in medication at diagnosis of UR | Attitude following the discovery of UR |
| --- | --- | --- | --- |
| Patient 1  Male  20 years old  66 kg | Crying, flexed trunk position,  worsening of auto-aggressive (punches to his abdomen, his thighs, his head) and hetero-aggressive challenging behaviors | -Clozapine (+)  - Stop guanfacine, bipéridène | - Urinary catheter (<24 hours)  -Toilet protocol  -Limitation of drugs at risk of UR (antipsychotic…)  - Laxatives  - Tamsulosin (long-term) |
| Patient 2  Female  27 years old  62 kg | Screams, worsening of auto-aggressive (banging her head against the walls, bites on her arms) and  hetero-aggressive challenging behaviors,  appearance of throwing water on her abdomen behavior | -Haloperidol (+)  -Levomepromazine (+)  -Venlafaxine (+)  -Lithium (+)  -Diazepam (+)  -Stop aripiprazole clonazepam trazodone gabapentine, hydroxyzine | -Toilet protocol  -Limitation of drugs at risk of UR (antipsychotic…)  - Laxatives |
| Patient 3  Male  25 years old  53 kg | Screams, crying, worsening of auto-aggressive (strokes of his hands towards his face) and hetero-aggressive challenging behaviors,  more than 6 hours without urine | -Olanzapine (+)  -Clobazam (+) | - Urinary catheter (<48 hours)  -Toilet protocol  -Limitation of drugs at risk of UR (antipsychotic…)  - Laxatives  - Tamsulosin (temporary) |
| Patient 4  Male  25 years old  55 kg | Appearance of auto-aggressive challenging behaviors (abdomen pinches, punches to his head),  appearance of behavior of showing and touching his penis, and of undressing, more than 6 hours without urine or urinary incontinence (overflow) | -Stop antipsychotics  -Stop antidepressants  -Stop valproic acid  -Tetrabenazine (+) | - Urinary catheter (in out)  -Toilet protocol  -Limitation of drugs at risk of UR (antipsychotic…)  - Laxatives  - Tamsulosin (long-term) |
| Patient 5  Male  18 years old  49 kg | Screams, crying, persistence of auto- (banging his head against the walls, bites of his arms) and hetero-aggressive challenging behaviors, more than 6 hours without urine | -Pregabalin (+)  -Tramadol (+)  -Stop Sertraline | -Toilet protocol  -Limitation of drugs at risk of UR (antipsychotic…)  - Laxatives |

*(+) = Introduction or dose increase; Toilet protocol = accompany the patient to the toilet every three hours and encourage him to urinate to empty his bladder*

**Supplementary Table 3***.* Summary of clinical manifestations and management of UR for the five participants

| Clinical manifestations identified during an episode of UR: | Management of UR: |
| --- | --- |
| More than 6 hours without urine (3/5) | Toilet protocol (5/5) |
| Crying, screaming (4/5) | Limitation of drugs (antipsychotic, etc.) (5/5) |
| Pain avoidance behavior e.g. flexed trunk, undressing, throwing water on his abdomen, touching his penis (3/5) | Laxatives (5/5) |
| Auto-aggressive behavior towards abdomen (2/5) or other areas e.g. head (5/5) | Urinary catheter (<48 hours) (3/5) |
| Hetero-aggressive behavior (4/5) | Tamsulosin (3/5) |
| Secondary enuresis (1/5) |  |
